# Trkb-IP3 Pathway Mediating Neuroprotection in Rat Hippocampal Neuronal Cell Culture Following Induction of Kainic Acid

**DOI:** 10.21315/mjms2018.25.6.4

**Published:** 2018-12-28

**Authors:** Pei Nei Chong, Muthuraju Sangu, Tee Jong Huat, Faruque Reza, Tahamina Begum, Abdul Aziz Mohamed Yusoff, Hasnan Jaafar, Jafri Malin Abdullah

**Affiliations:** 1 Department of Neurosciences, School of Medical Sciences, Universiti Sains Malaysia, 16150 Kubang Kerian, Kelantan, Malaysia; 2 Department of Pathology, School of Medical Sciences, Universiti Sains Malaysia, 16150 Kubang Kerian, Kelantan, Malaysia; 3 Centre for Neuroscience Services and Research, Universiti Sains Malaysia, 16150 Kubang Kerian, Kelantan, Malaysia

**Keywords:** rat hippocampal neuron culture, IP_3_ & BDNF-TrkB receptor, GABA_A_ receptor, kainic acid, epilepsy

## Abstract

**Background:**

Following brain injury, development of hippocampal sclerosis often led to the temporal lobe epilepsy which is sometimes resistant to common anti-epileptic drugs. Cellular and molecular changes underlying epileptogenesis in animal models were studied, however, the underlying mechanisms of kainic acid (KA) mediated neuronal damage in rat hippocampal neuron cell culture alone has not been elucidated yet.

**Methods:**

Embryonic day 18 (E-18) rat hippocampus neurons were cultured with poly-L-lysine coated glass coverslips. Following optimisation, KA (0.5 μM), a chemoconvulsant agent, was administered at three different time-points (30, 60 and 90 min) to induce seizure in rat hippocampal neuronal cell culture. We examined cell viability, neurite outgrowth density and immunoreactivity of the hippocampus neuron culture by measuring brain derived neurotrophic factor (BDNF), γ-amino butyric acid A (GABA_A_) subunit α-1 (GABRA1), tyrosine receptor kinase B (TrkB), and inositol trisphosphate receptor (IP_3_R/IP3) levels.

**Results:**

The results revealed significantly decreased and increased immunoreactivity changes in TrkB (a BDNF receptor) and IP_3_R, respectively, at 60 min time point.

**Conclusion:**

The current findings suggest that TrkB and IP3 could have a neuroprotective role which could be a potential pharmacological target for anti-epilepsy drugs.

## Introduction

Epilepsy is a brain disorder initiated by the perturbation of ionic equilibrium which results in neuronal hyper-excitability that causes unprovoked and recurrent seizure. This is a common neurological disorder, and various factors such as brain trauma, infection, and genetic factors contribute to its pathogenesis (1, 2). Seizures in adults and children often affect the hippocampal formation with distinct neuropathological features, including neuronal death, neurogenesis, gliosis, axonal sprouting and reorganisation of neuronal interconnections (3, 4). These abnormalities typically develop over weeks to years of epileptogenic events, and can occur in healthy tissue after an initial epileptogenic event, such as status epilepticus (5, 6).

Seizures can be divided into two types: generalised seizures and focal seizures. Generalised seizures involve both hemispheres, whereas focal seizures occur in specific areas of the brain. Seizures can be further divided according to the duration and location of the seizure (7), and epilepsy can be categorised on the basis of a patient’s age. Epilepsy with earlier onset is typically more detrimental, usually involving ionic changes in γ-amino butyric acid A (GABA_A_) receptors and changes of the balance between excitation and inhibition in the brain (8). In contrast, epilepsy in adults often exhibits a partial form, and its primary causes are trauma, injury and tumors (9). However, there are detrimental effects in the hippocampus whenever seizures occur.

To examine the factors underlying epileptogenesis, a number of experiments have been conducted using laboratory simulations in vivo, seeking to replicate the features of a human system. However, neurons exist in the mammalian brain over a subject’s lifetime. Thus, long-term cell culture may provide a useful method for mimicking mammalian physiological processes under laboratory culture conditions (10). Moreover, neuron culture models have been used to elucidate the molecular mechanisms underlying the development of neuronal polarity and dendritic growth and synapse formation by visualising the subcellular localisation of endogenous protein trafficking (11). Several types of seizure model are widely used, including chemoconvulsant and electrical kindling models. Pilocarpine and kainic acid (KA) are chemoconvulsant seizure models that can be tested in vitro. Pilocarpine-treated animals exhibit similar electroencephalographic features and neuropathological changes to KA-treated animals (12). Neuronal, glial and vascular lesions in Pilocarpine and Kainate models of Temporal lobe epilepsy (TLE) has been described in an article of Curia et al. (13). In previous studies, pilocarpine has mainly been applied via systematic injection, and the hippocampus is often the onset zone, as in the KA model (12). Importantly, the effects of KA have been validated, contributing to the understanding of the molecular, cellular and pharmacological mechanisms underlying epileptogenesis and ictogenesis (14).

The KA-induced model has been used to induce epileptogenic symptoms in vivo and in vitro, causing seizure, behavioural changes, oxidative stress, and production of inflammatory mediators (15). In the current experiment, KA was selected because of its validated simulation of pathological effects, with similar characteristics to the pathology of epilepsy and ischemia (16, 17). KA has been widely used as a model of epilepsy in both neuronal culture and in vivo models (12, 18). In addition, it has been reported that epileptogenesis can be caused by changes in homeostatic mechanisms involving calcium (18). Overloading of glutamate receptor activation by KA receptors induces an influx of cellular Ca^2+^, production of reactive oxygen species (ROS) and mitochondrial dysfunction (19, 20). Subsequently, Ca^2+^ sequestration mechanisms cause neuronal insult and neuronal death, including, hypoxia, ischemia, and epileptic seizures (14, 18, 19, 20). Importantly, KA was found to trigger neurogenesis, reporting that abnormal maturation of dendrites resulted in the occurrence of seizures. Neurogenesis in hippocampal regions was also found to be related to epileptogenesis (21).

Several studies have reported that brain derived neurotrophic factor (BDNF) has neuroprotective features, and BDNF mRNA expression has been found to increase after KA exposure (19, 22, 23). Interestingly, BDNF transgenic mice exhibited reduced seizure behaviour in response to KA under conditions of overexpression of truncated TrkB receptors (24). In addition, Koyama et al. reported the development of hyper-excitable circuits caused by activity-induced BDNF release and Trk receptor activity. These findings suggested that BDNF enables synaptic plasticity and subsequently elevates N-methyl-D-aspartate receptor (NMDAR) activity via exercise (25). As an exercise-induced effect, the accumulation of ketone bodies (D-β-hydroxybutyrate; DBHB) in the hippocampus serves both as an energy source and an inhibitor of class I histone deacetylases (HDACs) to specifically induce BDNF expression (25). In addition, the induction of BDNF expression results in neurogenesis, axonal and dendritic sprouting, and enhanced long-term potentiation (LTP) induction in a TrkB-dependent manner. However, this process can cause hyper-excitability in neurons, eventually resulting in excitotoxicity (26). Thus, a self-sustaining cycle of sprouting and hyper-excitability is partially fueled by activity-dependent BDNF release, and Trk receptors can precipitate the activation of phospholipase C (PLC), phosphoinositide 3-kinase (PI3K), and Ras, enabling neuronal survival and neurite outgrowth (27). BDNF regulates synaptic transmission and activity-dependent plasticity, as well as promoting LTP (26, 27). BDNF-TrkB effects on LTP occur through both pre-and postsynaptic mechanisms, exerting a presynaptic effect in a retrograde manner and a postsynaptic effect by modulating NMDA receptor subunit expression (28).

During early development of the nervous system, BDNF is also reported to be an essential modulator at postsynaptic sites, including the γ-aminobutyric acid (GABA) type A receptor (GABA_A_R)-mediated signaling that functions in parallel to the neurotrophin/tropomyosin-related kinase (Trk)-dependent, (also known as tyrosine receptor kinase) signaling involved in cell proliferation and migration (29, 30). As study demonstrated that the TrkB modulating synaptic structure mediates signaling in the hippocampus, reporting that BDNF treatment resulted in an increase in the number of NMDAR and GABA_A_R clusters, and increased the proportion of clusters opposed to the presynaptic terminal (31). Importantly, BDNF increased the clustering of GABA_A_R under conditions in which TrkB was blocked. TrkB exerts extensively mediated effects on postsynaptic neurotransmitters, to balance out inhibitory and excitatory synaptic transmission as neuronal circuits develop. This issue has been further investigated, revealing a GABAergic synapse deficit in mice with deleted TrkB in the cerebellum, with no changes in the number of glutamatergic synapses (32, 33).

Particularly in inhibitory GABA_A_R function, BDNF has been found to enhance synaptic responses and facilitate the induction of LTP by eliminating dendritic shunting and eventually promoting transduction of output signals (34). Researchers have consistently observed the overexpression of BDNF-induced enlargement of soma in GABAergic neurons in cultured E-18 Wistar rat hippocampal neurons. Previous authors have proposed that elevation of GABA_A_ and glutamic acid decarboxylase (GAD, GABA synthesising enzyme) facilitates high levels of K^+^ and elicits the release of GABA (35, 36). This suggests that BDNF principally promotes GABAergic maturation, but potentially contributes to excitatory synapse development via increasing resting synaptic vesicles. BDNF treatment has consistently shown an increment of GABA-current amplitude, which is elicited by GABA_A_ in hippocampal culture (37). In accord with this notion, decreased GABA_A_R function in hippocampal regions was found in a patient with medial temporal lobe epilepsy (MTLE) (38).

BDNF and its receptor TrkB are expressed at a relatively high level in the hippocampus and cerebral cortex, exerting a strong effect on downstream signaling that is critical for epileptogenesis (39, 40). The TrkB receptor has three downstream signaling pathways involving BDNF binding: phosphatidyl (PI3K), phopholipase Cγ (PLCγ) and mitogen activated protein kinases/extracellular signal-regulated kinases (MAPK/ERK), these pathways can lead to neurogenesis and synaptic plasticity, as suggested in previous studies (33, 41, 42, 43). However, it has been proposed that the PLCγ pathway may be involved in epileptogenic seizure (44). One of the products of PLCγ, inositol 1, 4, 5 trisphosphate (IP3) has the ability to mobilise intracellular Ca^2+^ (34, 44, 45, 46). Several studies have reported that the mobilisation of calcium ions suppresses GABA_A_R function, resulting in epileptogenic seizure (29, 37, 41). Moreover, application of KA to hippocampal neuronal culture is speculated to elevate the production of free radicals in the intracellular Ca^2+^, which specifically triggers production of IP3 and PLC_γ_, eventually affecting IP3R and likely leading to suppression of GABA_A_R because of the over-excitation of GluR5 receptors (kainate receptors). However, it remains unclear why IP3 is an indicator of the suppression of GABA_A_R and downstream BDNF-TrkB.

In the present study, GABAR, BDNF, TrkB, IP3, and IP3R were assessed to investigate the difference of these receptors in normal and epileptogenic hippocampal neuron culture. These receptors were highlighted as important components with significant responses to KA-induced seizure in previous research (34, 39, 40, 44).

Therefore, in the current study we examined changes in GABA_A_ receptors, BDNF-TrkB receptors and IP3 receptors in normal and epileptogenic primary rat hippocampal neuron culture at embryonic day 18 (E-18), to elucidate the mechanisms of epileptogenic seizure and provide insights for anti-epilepsy drug (AED) development.

## Material and Methods

### Cell Culture

Primary rat hippocampal neuron (PRHN; A104841-01) was purchased from Gibco™, USA. PRHN was isolated from day-18 Fischer 344 rat embryos, then cultured using the Neurobasal®-medium (Gibco™, 21103049) supplemented with GlutaMAXTM-I Supplement (Gibco™, 35050061) and B-27® Serum Free Supplement (Gibco™, 17504044) with proportions based on the protocol provided by the manufacturer. In addition, L-glutamine (Gibco™, 25030081) was supplemented for the first 4 days of culturing in accord with the manufacturer’s suggested protocol. The PRHN was plated with a neuronal density of 900 cells/mm^2^ which have a maturity rate that suits our experiment design (45). PRHN was cultured on a culture plate coated with 0.01% poly-L-lysine (Sigma-Aldrich, 25988-63-0): molecular weight-70000–150000 according to the coating method described by Todd et al. (10). The cells were cultured in incubator at a temperature of 36 °C–38 °C with 5% CO_2_. The culture was maintained, aspirating half of the media from each well and replacing it with fresh composite media every third day.

### Kainic Acid Treatment

KA (Sigma-Aldrich, 58002-62-3) 10 mg was added to 1 mL of Dulbecco’s phosphate-buffered saline (DPBS) (Gibco™, 14040117). Serial dilution was then performed using DPBS (Gibco™, 14040117) to obtain 0.5 μM KA. Next, 0.5 μM KA was added to the cultured PRHN and incubated for 30, 60 or 90 min.

### Morphological Assessment

To examine the morphological changes of PRHN along the culturing period, we captured digital images of cultured cells in non-overlapping fields using phase contrast microscopy (Axiovert 200) to examine cell morphology for structural alterations in cell bodies, neurites and cellular interconnection.

### Cell Viability Testing

We used a neurite outgrowth staining kit (Invitrogen™, A15001) to examine the viability and health of cells. The PRHN was plated in a 96-well cell culture plate with 3.0 × 104 neurons per well, then stained with neurite outgrowth staining after KA treatment. Neurite outgrowth staining containing a cell viability indicator, and cell membrane staining, which emit green fluorescence and bright red orange fluorescence, respectively. The fluorescence intensity of the stained cells was assessed using a microplate reader VariosKan 2000 and SkanIt™ Software. Bottom-read fluorescence detection was performed using monochromator excitation/emission setting 483/525 nm (12 nm bandwidth) for green fluorescence cell viability indicator and 554/567 nm (5 nm bandwidth) for the orange-fluorescent cell membrane stain. Cell-free controls were included and used for background subtraction. Cell treatments were typically performed in triplicate, and the mean ± standard error of the mean (SEM) was plotted using GraphPad Prism 7™ software.

### Immunocytochemistry

To examine the immunoreactivity of BDNF, TrkB, GABRA1 and IP3, PRHN was plated in a 6-well plate with 25 mm round glass coverslips coated with poly-L-lysine. PRHN was plated with 1.8 × 105 cells in each well, and incubated at 36 °C–38 °C in a humidified atmosphere of 5% CO_2_ in incubator (10). The different treatment groups underwent cell fixation with 4% paraformaldehyde (PFA) for 15 min. The cells were rinsed with DPBS (with Ca^2+^ and Mg^2+^) twice and permeabilised with 0.3% Triton-X (diluted in DPBS with Ca^2+^ and Mg^2+^) for 15 min at room temperature. Following several washes in DPBS with Ca^2+^ and Mg^2+^, cells were incubated in blocking solution (5% goat serum solution in DPBS with Ca^2+^ and Mg^2+^) for 2 h at room temperature and incubated with primary monoclonal antibodies against BDNF (1:1000; Abcam, UK), TrkB (10:1000; Abcam, UK), GABRA1 (1:1000; Abcam, UK), IP3 (1:1000; Abcam, UK), respectively overnight at 4 °C. The following day, cells were washed several times in DPBS with Ca^2+^ and Mg^2+^. Goat anti-mouse IgG (H+L) highly cross-adsorbed secondary antibody conjugated with Alexa Fluor 488 (Life Technologies, USA) or goat anti-rabbit IgG (H+L) highly cross-adsorbed secondary antibody conjugated with Alexa Fluor 594 (Life Technologies, USA) diluted in antibody diluent was added at a ratio of 1:1000 and incubated at room temperature for 2 h. Cells were washed with DPBS with Ca^2+^ and Mg^2+^ for 5 min each, and a small drop of Prolong® Gold anti-fade reagent was added, cover slipped, and used for microscopic analysis. Microscopic images were captured using EVOS FL Auto 2 Cell Imaging System (Thermo Fisher Scientific, USA), and quantification of cell fluorescence intensity was carried out using ImageJ software (Version 1.46; NIH, USA) (47, 48). There were more than five figures taken to analysis each and every groups of neuronal cells. The figures were taken randomly in cell culture flask.

### Statistical Analysis

Two independent experiments were carried out in triplicate for cell viability analysis and immunocytochemistry. All data are presented as mean ± SEM. For statistical comparison between different treatment groups, Analysis of variance (ANOVA) with Bonferroni’s test was used. Significant differences are indicated by asterisks in the figures (*P < 0.05; **P < 0.001).

## Results

### Morphological Changes in Hippocampal Neuron Culture

**Figure 1:**
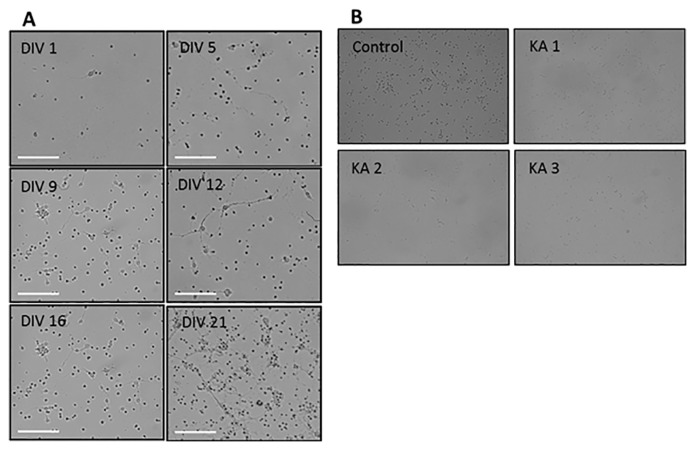
Morphological changes of E-18 rat hippocampal neuron culture. (A) Inverted microscopy images of hippocampal neuronal culture on different days-in-vitro (DIV) at 10× magnification using a Dino-Eye camera. A–F Morphological changes of the cell were observed at every 3 to 4 days of DIV 1; DIV-5; DIV 9; DIV-12; DIV 16; DIV 21. Scale bar represents 200 μm; (B) Inverted microscopy images of hippocampal neuronal culture at DIV12 at 10× magnification using a Canon Ds126191 microscope. Morphological changes of the cell were observed and captured in the neuron culture without treatment as a control condition, and culture with KA treatment as the treatment conditions (KA1: 30 min KA treatment; KA2: 60 min KA treatment; KA3: 90 min KA treatment). Scale bar represents 200 μm

Changes in hippocampal neuron culture, such extension of dendrites and the connectivity of neurons, can be observed with light microscopy. The captured images are shown in Figure 1. E-18 hippocampal neurons were cultured for up to 21 days, and images were captured every 3 days to observe morphological changes. The results revealed that cells began to emit neurites after a few hours of plating. Neurites emerged gradually from the dark spot representing the neuron, observed under 10× magnifications with an inverted microscope. In addition, we observed changes in the culture media using phenol red as indicator of change from pinkish-red media to golden culture media after the first 24 h of incubation. At DIV 1 onward, the sprouting of dendrites became denser as the culture continued, and the connections of neurons became more intense. The cellular stages of maturity are indicated by dendritic and axonal formation (49). At DIV 21, the network of dendrites was complex, and the cells were mature. These results are in accord with previous cell culture studies in which differentiated neurons developed extensive axonal and dendritic arbors and formed numerous functional connections with one another (10, 11, 47).

### Morphological Changes of E-18 Rat Hippocampal Neuronal Culture Following KA Exposure

To examine the time course and localisation of KA-induced neuronal damage in vitro, hippocampal neuron cultures were treated with 0.5 μM of KA for various times (30 min, 60 min and 90 min) at the peak of neuron characterisation DIV-12 (47). As shown in Figure 1B, neurite extension and the networking of dendrites were reduced compared with the control group. After 90 min of KA exposure in hippocampal neuron culture, the floating debris in the media increased and the shape of the neurons indicated shrinking with truncated dendrites. The flotation of debris is a sign of neuronal detachment from the culture surface due to a loss of adhesion to the culture floor.

### Cell Viability and Neurite Outgrowth Density

**Figure 2:**
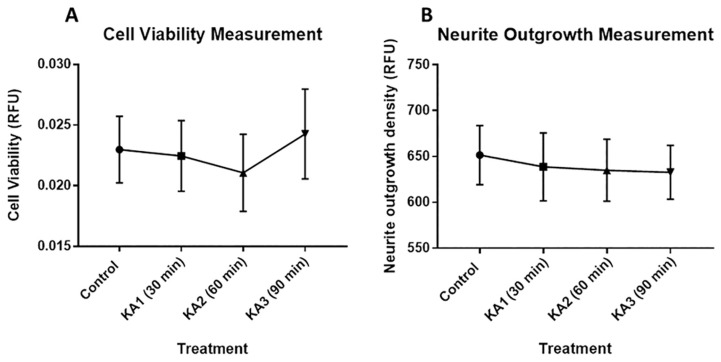
(A) Measurement of cell viability in RFU units for the control group and treatment groups (KA1, KA2 and KA3, incubated with 0.05 μm KA for 30, 60 and 90 min, respectively). (B) Neurite outgrowth density in RFU units in the control group and treatment groups (KA1, KA2, and KA3, incubated with 0.05 μm KA for 30, 60 and 90 min, respectively)

To identify changes in neurons following KA exposure, we quantified cell viability and neuronal outgrowth of living cells using the cell viability indicator in the neurite outgrowth kit (Thermo Fisher Scientific, US). The average intensity of cell viability labeling showed no significant differences (F [3, 32] = 1.585, *P =* 0.2123; α = 0.05). However, the graph of cell viability (RFU) of neuronal culture (Figure 2A) showed a trend towards different cellular responses to KA treatment for 30, 60 and 90 min, compared with the control group. KA1 showed no changes of cell viability compared with controls (0.0222 ± 0.0029, *P >* 0.99), while cell viability of the cell incubator for 60 min (KA2) showed a mild decrement (0.0211 ± 0.0032, *P >* 0.05). In contrast, cell viability of KA3 showed a slight increase (0.0242 ± 0.0037, *P >* 0.05).

We conducted another assessment using the neurite outgrowth kit (Thermo Scientific, USA), which provides a measure of neurite outgrowth density (RFU units). The average neurite outgrowth density showed no significant effects in the neuronal culture after 30, 60 and 90 min of KA incubation (F [3, 32] = 0.5719, *P =* 0.6376, α = 0.05). Although there were no significant effects, Figure 2B shows a trend towards a decrement of neurite density over time. In addition, the data indicated a slight but non-significant reduction in neurites of the cell as the incubation time increased: KA1 (638.7 ± 37.1, *P >* 0.05); KA2 (634.7 ± 33.74, *P >* 0.05); KA3 (632.8 ± 29.35, *P >* 0.05). Although this decrement was not significant, the trend suggests that KA may have affected the neurite or dendrite extension in the cell, reducing the level of fluorescence emitted.

### Expression of BDNF, TrkB, IP3 and GABRA1

**Figure 3:**
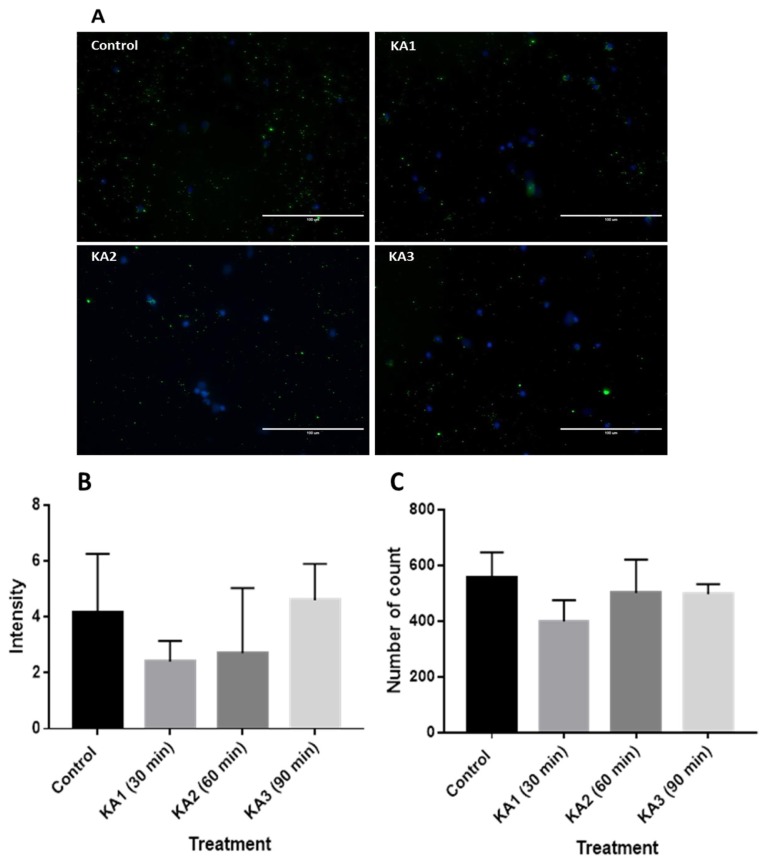
(A) Immunofluorescence images of hippocampal neuronal culture stained with primary antibody: anti-BDNF (Abcam) and secondary antibody Alexa Fluor 488 (Invitrogen) co-stained with Dapi (Invitrogen) for control; KA1: hippocampal neuronal culture + KA incubated 30 min; KA2: hippocampal neuronal culture + KA incubated 60 min; KA3 hippocampal neuronal culture + KA incubated 90 min. The scale bar represents 100 μM. (B) Intensity of BDNF stained rat hippocampal neuronal culture with control group (without KA), KA1 (30 min KA incubation), KA2 (60 min KA incubation), KA3 (90 min KA incubation). (C) Number of BDNF stained particles count in rat hippocampal neuronal culture with treatment: control group (without KA), KA1 (30 min KA incubation), KA2 (60 min KA incubation), KA3 (90 min KA incubation)

**Figure 4:**
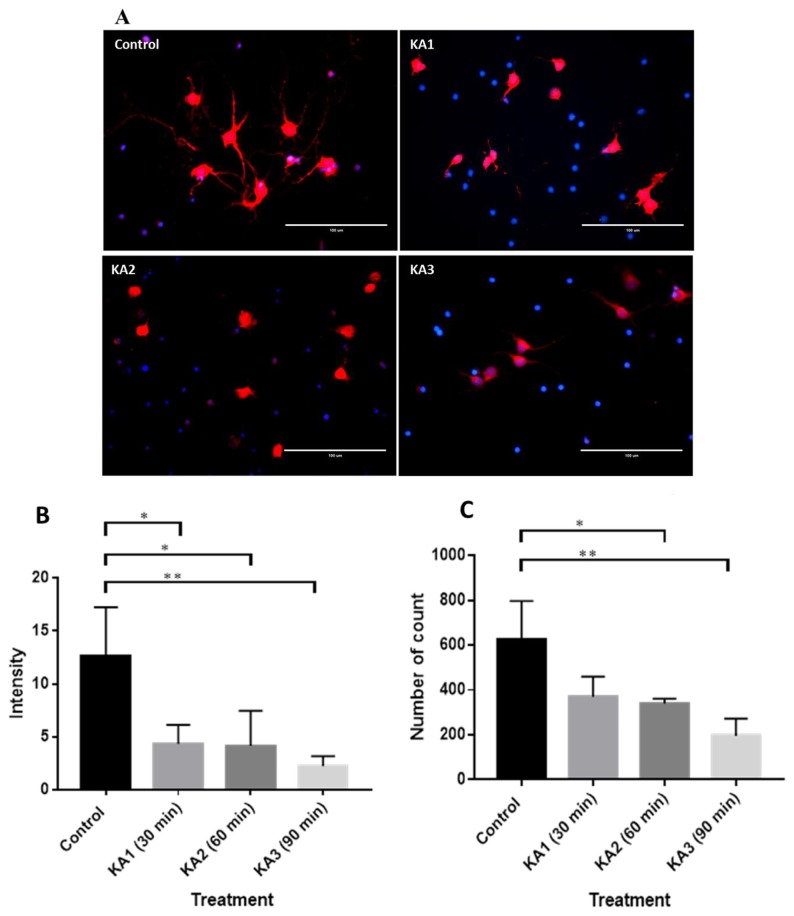
KA inhibited TrkB expression. (A) Immunofluorescence images of hippocampal neuronal culture stained with primary antibody: Anti-TrkB (Abcam) and secondary antibody Alexa Fluor 488 (Invitrogen) co-stained with Dapi (Invitrogen). (B) Immunofluorescence intensity from control and cell culture with KA treatment; KA1: 30 min KA treated neuron culture; KA2: 60 min KA treated neuron culture; KA3: 90 min KA treated neuron culture. (C) Number of TrkB stained particles count in rat hippocampal neuronal culture with treatment: control group (without KA), KA1 (30 min KA incubation), KA2 (60 min KA incubation), KA3 (90 min KA incubation)

**Figure 5:**
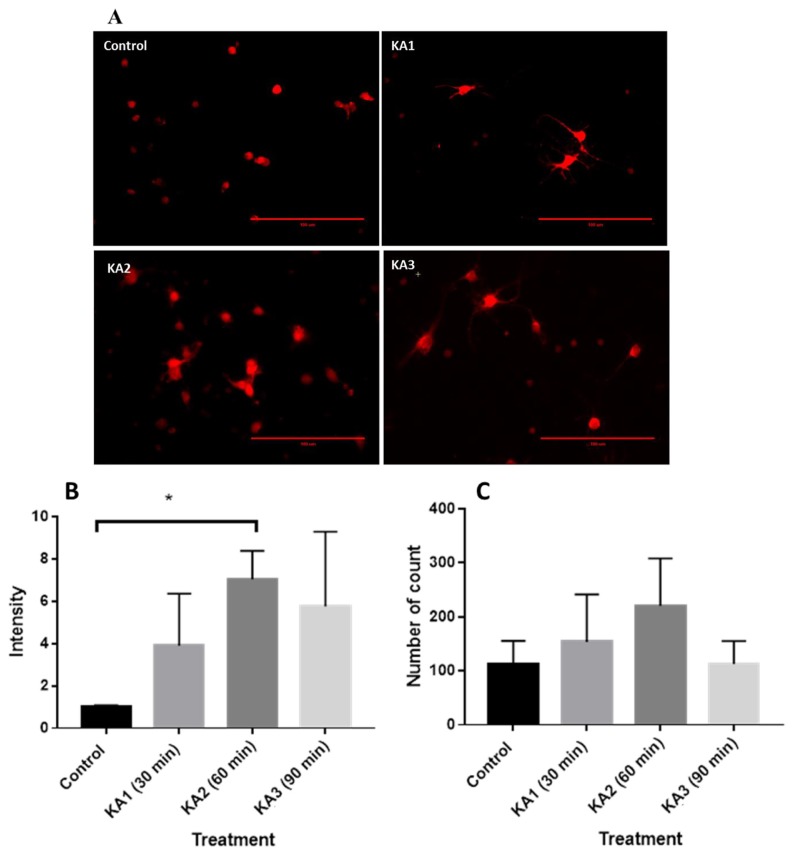
IP3R induced after KA introduction. (A) Immunofluorescence images of hippocampal neuronal culture stained with primary antibody: Anti-IP3R (Abcam) and secondary antibody Alexa Fluor 594 (Invitrogen) (B) Immunofluorescence intensity from control and cell culture with KA treatment; KA1: 30 min KA treated neuron culture; KA2: 60 min KA treated neuron culture; KA3: 90 min KA treated neuron culture. (C) Number of IP3 stained particles count in rat hippocampal neuronal culture with treatment: control group (without KA), KA1 (30 min KA incubation), KA2 (60 min KA incubation), KA3 (90 min KA incubation)

**Figure 6:**
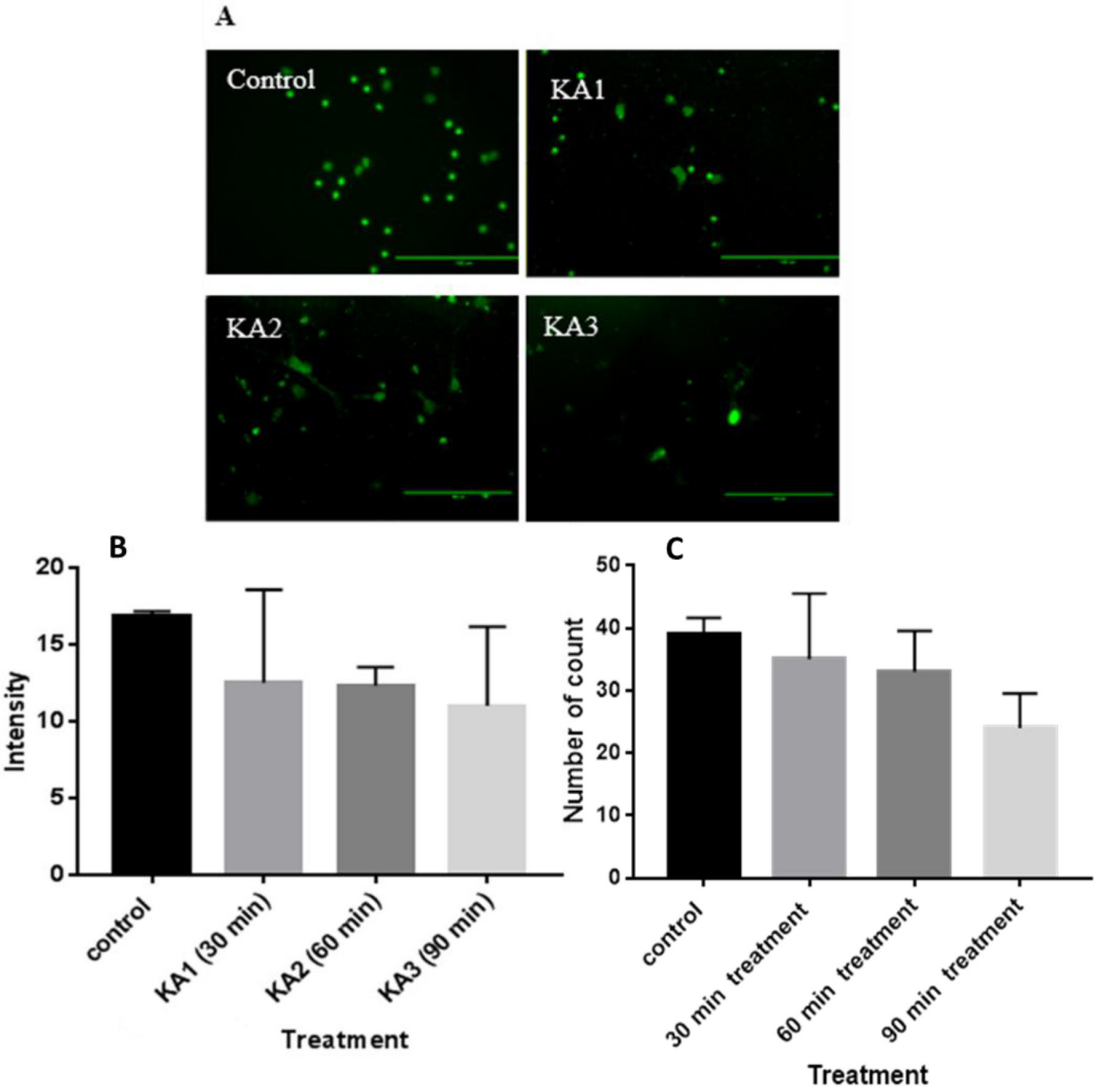
KA did not significantly affect GABA_A_R α-1. (A) Immunofluorescence images of hippocampal neuronal culture stained with primary antibody: anti-GABRA1 (Abcam) and secondary antibody Alexa Fluor 488 (Invitrogen) Control; KA1: hippocampal neuronal culture + KA incubated for 30 min; KA2: hippocampal neuronal culture + KA incubated for 60 min; KA3: hippocampal neuronal culture + KA incubated for 90 min. The scale bar represents 100 μM. (B) Intensity of GABRA1 stained rat hippocampal neuronal culture in the control group (without KA), KA1 (30 min KA incubation), KA2 (60 min KA incubation), KA3 (90 min KA incubation). (C) Number of GABA_A_R α-1 stained particles count in rat hippocampal neuronal culture with treatment: control group (without KA), KA1 (30 min KA incubation), KA2 (60 min KA incubation), KA3 (90 min KA incubation)

Immunostaining was conducted in the normal and epileptogenic hippocampal neuronal culture. BDNF and GABRA1 receptors were stained with Alexa Fluor 488, which emits green light when the fluorescence beam is bombarded. BDNF was found to be located extracellularly, scattered around the cell as shown in Figure 3A. Meanwhile, anti-TrkB (Figure 4A) and anti-IP3 (Figure 5A) were stained with red using Alexa Fluor 593. In addition, anti-GABRA1 receptors (Figure 6A) were also found in the neurons in which green was expressed in the image.

### Expression of BDNF Following KA Treatment

KA treatment-related changes in BDNF immunofluorescence intensity were quantified from the images (Figure 3A) of the hippocampal neuronal culture and compared with KA non-treated cell culture as a control. The level of the BDNF signal was not significantly influenced by KA treatment at 30, 60 and 90 min (F [3, 8] = 1.145, *P >* 0.05). However, inhibition was observed at KA1 (2.409 ± 0.7357, *P >* 0.05) and KA2 (2.712 ± 2.325, *P >* 0.05) followed by an increment at KA3 (4.603 ± 1.299, *P >* 0.05) (Figure 3B).

### Expression of TrkB Following KA Treatment

KA treatment-related changes in TrkB immunofluorescence intensity were quantified from the images (Figure 4A) of the hippocampal neuronal culture and compared with KA non-treated cell culture as a control. The level of TrkB fluorescence intensity was significantly decreased by KA treatment at 30, 60 and 90 min (F [3, 8] = 1.145, *P =* 0.010, α = 0.05). The number of TrkB particles at KA1 (30 min of KA incubation) and KA2 (60 min of KA incubation) exhibited a slight non-significant decrease (370.3 ± 89.63, *P =* 0.050), and a significant decrease (340.3 ± 20.82, *P =* 0.030*), respectively. At 90 min of KA incubation, KA3 exhibited a significant reduction of particles (195.7 ± 76.7, *P <* 0.01**). The TrkB immunofluorescence intensity (Figure 4B) and the number of particles (Figure 4C) are correlated.

### Expression of IP3R Following KA Treatment

As shown in Figure 5A, KA treatment-related changes in IP3R immunofluorescence intensity were quantified from images in the hippocampal neuronal culture, and compared with KA non-treated cell culture as a control. The IP3R signal was significantly affected by KA treatment (F [3, 8] = 6.954, *P =* 0.049, α = 0.05). IP3R was significantly expressed at KA2 (7.045 ± 1.338, *P <* 0.05). The results indicated an increment of IP3R, suggesting that downstream BDNF, TrkB and/or GABRA1 were triggered in the KA-treated neuron culture. The IP_3_R immunofluorescence intensity (Figure 5B) and the number of particles (Figure 5C) are correlated except at KA3.

### Expression of GABA_A_R α-1/GABRA1 Following KA Treatment

The KA treatment-related changes in GABRA1 immunofluorescence intensity were quantified from the images (Figure 6A) of the hippocampal neuronal culture, and compared with KA non-treated cell cultures as a control. The level of the GABRA1 signal was not significantly influenced by KA treatment at 30, 60 and 90 min (F [3, 8] = 1.178, *P =* 0.372; α = 0.05) as shown in Figure 6B. However, although no significant decrement was found, the imaging data indicates a trend towards declining intensity in images captured from immune-stained neuron cultures. The GABRA1 immunofluorescence intensity (Figure 6B) and the number of particles (Figure 6C) are correlated.

## Discussion

In the present study, to reveal the time course and localisation of KA-induce neuronal damage in vitro, cultured hippocampal neurons were treated with 0.5 μM of KA at various times (30 min, 60 min and 90 min), followed by neurite outgrowth and cell viability assay. The neurite outgrowth cell viability assay is considered important for examining the morphological phenotype of neuron culture and cell health (50). Moreover, this staining kit also determines neurite outgrowth density, rapidly isolating rat cortex neurons and iPSC-derived iCell neurons and PC-12 derived neuroscreen-1 cells, using an unbiased microplate reader with two different coloured dyes (cell membrane stain and cell viability indicator) and a background suppression dye (50). This method is faster than a previous method for assessing neurite outgrowth density by immunocytochemical staining (beta-III tubulin or MAP2) followed by manual or automated microscopic image acquisition and analysis (51, 52). However, this immunocytochemistry technique is still required for identifying the morphological features, particularly parameters such as neurite length, soma size and neurite complexity (53).

The present study demonstrated that KA treatment induced a neurodegenerative effect in embryonic rat hippocampal neuron culture. Morphological changes were detected, including disrupted dendritic arbor development and truncated dendrites after KA incubation. These are early indicators of neurodegeneration, demonstrating that neurons had begun to lose dendrites, cell bodies appeared to shrink, and cytoplasmic material was lost and detached from neighboring cells or the culture surface (54, 55). Previous studies suggested that KA caused damage to the cell body and degeneration in the nucleus, causing darkening in color and truncated dendrites as early cellular changes in response to toxic insult (56). However, in the current study, there was significant difference in cell viability and neurite outgrowth density of the hippocampal neuron culture between normal and KA-treated neurons at 60 min whereas compared with 90 min did not. The underlying mechanisms need to be elucidated.

Transformations from excitatory to inhibitory activity were observed in the present study. Quantification of immunoreactivity was not detected in particle count, possibly because of the transformation of excitatory to inhibitory activity in GABRA1 in the maturation of neurons. Alterations of excitatory and inhibitory interneurons are substantially diminished in the sclerotic hippocampus and following status epilepticus (SE) seizures (55, 57). During the early stages after SE, dramatic changes occur in the hippocampus, including cerebral edema and compensatory changes, as demonstrated by 1H-MR spectroscopy soon after injury in vivo. An early decrease of glutamate levels and increased GABA levels at 8 days onwards indicates progressive development of hippocampal neurodegeneration (57). Studies of the effects of elevated BDNF in GABAergic neurons have indicated that BDNF decreases GABAergic transmission and decreases K-Cl transporter (KCC2) activity (58). Upregulation of GAD-expressing interneurons has been identified after KA (59). Compensatory mechanisms occur before refractory seizure because of upregulation of GAD67 in granule cells (56). In developing neurons, protein tyrosine kinases are essential in mediating the switch of GABAergic inhibition, and disruption of a specific subtype of KCC2 has been found to increase seizure susceptibility in young and adult animals (60). Loss of GABAergic neurons inhibits neuronal excitability by downregulating the BDNF-TrkB signaling pathway after practicing treadmill exercise in epileptic rats.

Moreover, a study using a theta-burst stimulation (TBS)-induced model reported that activation of metabotropic glutamate receptors (mGluRs) is involved in KCC2 in cortical neurons, leading to increased intracellular Ca^2+^ (61). GABA_A_R may also be affected by the GluR5 kainate receptor after KA treatment. GluR5 is one of the kainate receptors located in the CA1 and CA3 subfields (14, 62). One previous study revealed that GluR5 knockout mice exhibit increased epileptogenic activity, whereas knockout of GluR6 prevents the induction of epileptogenic activity (63). In addition, upregulation of GluR5 mRNA levels occurs in the hippocampus when KA is administered (63). Thus, the decrease of GABRA1 could be the result of upregulation of mGLuR5 after KA treatment. A review of GABAR and mGluR5 signaling in the cerebellum in patients with schizophrenia, mood disorders, and autism reported that GABRA1 is affected by mGluR5 (64). However, molecular cascades should be studied in more depth to elucidate the relationship between mGLUR5 and GABRA1. A study reported that the function of GABAergic neurons is usually conserved in absence epilepsy and GABAergic inhibition is considered to potentiate clinical and experimental seizure initiation (66). The different role of GABAA and GABAB receptors reported that spike and wave discharges (SWD) related activity occurred without significant contribution of GABAB receptors but inhibit of GABAA receptors potentiates SWD related activity (67). GABAA and GABAB receptors activation differently regulated spindle like rhythmic activity in seizure (68). Another previous study demonstrated that rhythmic inhibitory potentials through GABAA but not GABAB receptor activation during seizure in thalamocortical neurons (69).

BDNF and its receptors have been implicated in the development of epilepsy. Conditioned media can protect hippocampal neurons against KA-induced excitotoxicity, stimulating endogenous cell survival factors BDNF and anti-apoptotic Bcl-2 in the host cell (19). In addition, increased seizure activity has been found to upregulate BDNF mRNA and protein expression in immunoreactivity, and to activate TrkB in the epileptic brain (22, 28, 36, 65, 66). BDNF knockout mice have been reported to exhibit overexpression of TrkB in response to KA (24, 67). BDNF signal modifies epileptogenesis, as reviewed by Lähteinen et al. (72), however, previous studies reported that BDNF showed no significant change in immunoreactivity in KA-treated hippocampal neuron culture. In addition, p75NTR and proBDNF would be expected to act as prior upregulated proteins in KA-treated neurons because they are released in apoptosis and necrosis after seizure or TrkB receptor binding and activation of ERK, and neurite outgrowth is reported to cease after prolonged p75NTR activation and apoptosis in neurons (28, 58). Upregulation of p75NTR was found in the hippocampus following chemoconvulsant seizure (using KA and pilocarpine), with elevation of immunoreactivity in neurons and astrocytes (73, 74). The seizure activity increases the excitability of primary cells, increasing the likelihood of epileptogenesis via morphological changes in dendritic spine, and stimulating axonal growth. This notion is supported by the finding of significant upregulation of BDNF and TrkB immunoreactivity in hippocampal neurons (in dentate gyrus and CA3) but no clear difference between patients with hippocampal sclerosis and those without hippocampal sclerosis (69). Turnover of hippocampal neurons rather than neurogenesis per se and BDNF signaling are required for the long-term survival of newborn neurons in the mouse hippocampus (57). One previous study reported that epileptogenesis was was initiated by excitability rather than being triggered by axonal spouting (27). This disparity is thought to indicate that chronic infusion of BDNF can downregulate TrkB receptors (75). In addition, downregulation of TrkB can ameliorate some conditions in which BDNF is elevated. However, this highlights the complexity of the issue, because BDNF does not always decrease TrkB. In addition, TrkB can be moved to a different cellular compartment, potentially playing new functional roles. Trk and p75 neurotrophic receptor (p75NTR)-containing vesicles are linked to axonal transport processes (76). Activation of secondary receptors, in which p75NTR modulates Trk receptor binding, mediates the activation of ERK from RAS, and neurite outgrowth activates c-jun N terminal kinase leading to apoptosis and triggering the secondary messenger MAP/ERK pathway, involving proto-oncogene c-fos and nitric oxide (NO)-producing neurons (77).

Our experiments consistently indicated that TrkB receptors were significantly reduced after KA was introduced into cultured neurons. We speculate that TrkB can cause transient inhibition to prevent recurrent seizure, exerting a neuroprotective effect. This thought is in line of the previous study that activation of TrkB protects neurons from dying (78). Eventually, the promotion of epilepsy by excessive TrkB kinase could also be prevented by uncoupling TrkB from the main signaling pathway of PLCγ1 (79). Phosphorylation of N-shC and PLCγ1 occurs after docking to Trk receptors, and PLCγ1 catalyses the breakdown to inositol 1,4,5 triphosphate, increasing Ca^2+^ in intracellular storage and DAG regulates protein kinase C (28). A study demonstrated that neuronal loss in the hippocampus by KA-induced epileptiform activity was mediated by the N-SHC signaling pathway (80). The current results suggest that IP3R activity is eventually affected, and that increased immunoreactivity of IP3R could be caused by intracellular Ca^2+^ release.

The upregulation of IP3R might have been caused by oxidative stress following KA treatment. Neuroexcitotoxicity properties were exhibited when KA was bound to kainite receptors (15). The overactivation of kainite receptors produces neuronal membrane depolarisation, causing excessive influx of Ca^2+^ into the neuron. In turn, this process disrupts the equilibrium of antioxidants and increases intracellular Ca^2+^ production of free radicals, resulting in oxidative stress in the neurons (15). The occurrence of oxidative stress suggests the presence of neuronal and glial death due to vulnerability of the brain in response to oxygen and glucose (15, 76). ROS are a mediator of oxidative stress, acting as oxidant agents that oxidise membrane lipids, protein, and DNA. As levels of ROS increase, a range of neurodegenerative agents also increases, including superoxide anions (O_2_^−^), hydrogen peroxide (H_2_O_2_) and highly reactive hydroxyl radicals (^·^HO) (76). Increases in these agents can lead to necrosis and apoptosis (81).

## Conclusion

In the present study concluded that KA induced neuronal damage within 30 min of exposure. The neuronal damage could mediate through TrkB-IP3 pathway as shown in the results. BDNF and GABA receptors did not show any significant changes as compared with control. In contrast, in the animal study reported that BDNF expression was increased after KA induction. As in the in vitro model with only neuron culture, BDNF did not change. The results suggested that the KA mediation of neuronal damage not dependent GABA or BDNF activation. The mechanism underlying TrkB-IP3 mediated neuroprotection need to be studied. However, the present finding strongly suggested that TrkB-IP3 could be important pathway to elucidate in future.
